# Rate of crop‐weed hybridization in *Sorghum bicolor* × *Sorghum halepense* is influenced by genetic background, pollen load, and the environment

**DOI:** 10.1111/eva.13536

**Published:** 2023-03-25

**Authors:** Cynthia Sias, Nithya Subramanian, George Hodnett, William Rooney, Muthukumar Bagavathiannan

**Affiliations:** ^1^ Department of Soil and Crop Sciences Texas A&M University College Station Texas USA

**Keywords:** crop‐wild hybridization, gene flow, herbicide resistance spread, novel trait confinement, outcrossing, technology stewardship, weedy relatives

## Abstract

The potential for gene flow between cultivated species and their weedy relatives poses agronomic and environmental concerns, particularly when there are opportunities for the transfer of adaptive or agronomic traits such as herbicide resistance into the weedy forms. Grain sorghum (*Sorghum bicolor*) is an important crop capable of interspecific hybridization with its weedy relative johnsongrass (*Sorghum halepense)*. Previous findings have shown that triploid progenies resulting from *S. bicolor* × *S. halepense* crosses typically collapse with only a few developing into mature seeds, whereas tetraploids often fully develop. The objective of this experiment was to determine the impact of *S. bicolor* genotype and pollen competition on the frequency of hybridization between *S. bicolor* and *S. halepense*. A total of 12 different cytoplasmic male sterile *S. bicolor* genotypes were compared with their respective male fertile lines across 2 years, to assess the frequency of hybridization and seed set when *S. halepense* served as the pollinator parent. Results indicate significant differences in the frequency of interspecific hybridization among the *S. bicolor* genotypes, and pollen fertility in *S. bicolor* reduced the rate of this interspecific hybridization by up to two orders of magnitude. Further, hybridization rates greatly varied across the two study environments. Results are helpful for developing appropriate gene flow mitigation strategies and indicate that gene flow could be reduced by the selection of appropriate seed parents for sorghum hybrids.

## 1 INTRODUCTION

Gene flow is an important evolutionary force in agricultural landscapes (Morrell et al., 2005; Slatkin, 1987) and is defined as, “the movement of gametes, individuals, and even entire populations” (Slatkin, 1987). In plants, gene flow occurs through the movement of pollen, seed, and/or vegetative propagules (Beckie et al., 2019). Gene flow is not equal to dispersal if the novel alleles are not incorporated into the gene pool (Mitton, 2013). Thus, in the context of this research, the process of gene flow does not only refer to the movement of alleles, but also successful hybridization resulting in the production of viable seed.

Gene flow between crop‐weedy/wild relatives in agricultural landscapes is expected to influence the evolutionary adaptation of weedy/wild types in several ways (Ellstrand, 1992; Ellstrand et al., 1999). Gene flow can help maintain or improve genetic diversity, especially that of small populations (Ksiazek‐Mikenas et al., 2019), and favor adaptation when exposed to selection (Smith et al., 2020; Sork, 2015). For example, Paterson et al. (2020) suggested that the spread of invasive johnsongrass (*Sorghum halepense* L. Pers.) may have been favored by introgression of adaptive traits from its crop relative, cultivated *S. bicolor*. High levels of gene flow are known to promote genetic homogeneity in weed populations across a landscape (Delye et al., 2010). Further, gene flow can enhance the fitness of weedy relatives at their range edges and contribute to further range expansion, especially under scenarios of climate change (Bontrager & Angert, 2018).

Crop‐wild gene flow, on the other hand, can lead to the replacement of wild genes by crop genes (called ‘genetic assimilation’ in the crop literature where wild genes are replaced by crop genes) and demographic swamping (hybrid progenies being less fertile than their wild parents). Using simulation models, Haygood et al. (2003) have shown that genetic assimilation can be fast even for disfavored crop genes, while demographic swamping can relax the conditions of genetic assimilation. Crop‐wild gene flow can also lead to the loss of wild population integrity (Sagnard et al., 2011) and valuable gene pools (Ellstrand, 2005). Cornille et al. (2013) have indicated that crop‐wild gene flow potential needs to be considered for in‐situ as well as ex‐situ conservation of plant genetic resources.

Crop‐wild gene flow occurs more frequently than previously thought (Ellstrand et al., 1999). In agricultural landscapes, weedy relatives of a number of cultivated crops [e.g., sorghum (*Sorghum bicolor* (L.) Moench), rice (*Oryza sativa* (L.)), sunflower (*Helianthus annuus* (L.))] coexist, and gene flow between them may lead to the transfer of adaptive traits to the weed species (Arias & Rieseberg, 1994; Ellstrand & Rieseberg, 2016; Shivrain et al., 2007). Since several novel transgenic and non‐transgenic traits have been incorporated into these crops, gene flow stands to become an important force in agricultural landscapes with significant evolutionary implications (Fernandez‐Cornejo & McBride, 2000). Sorghum is an important crop in the world and is a staple food crop in Africa and Asia (Rooney, 2004). In the United States, grain sorghum is produced on a total of 2.08 million hectares, with Kansas, Texas, Colorado, and Oklahoma being the lead production states (USDA, 2019). *S. bicolor* is primarily a self‐pollinating plant but can also receive pollen from nearby compatible sources. *Sorghum halepense*, a noxious wild/weedy relative of cultivated *S. bicolor*, is commonly found throughout the sorghum production areas in the world (Brown et al., 1988). *S. halepense* is a tetraploid (2n = 4× = 40) that shares a genome in common with cultivated *S. bicolor* (2n = 2× = 20) (Fernández et al., 2013). *Sorghum halepense* is a predominantly selfing species, but outcrossing can also be significant (Jhala et al., 2021; Maity et al., 2022). Because of their close relationship, interspecific hybridization has been reported between these two species (Arriola & Ellstrand, 1996; Hodnett et al., 2019).

Hybridization between *S. bicolor* and *S. halepense* may result in the transfer of novel traits, with significant agronomic and environmental consequences (Ohadi et al., 2017, 2018). Gene flow from *S. halepense* can impact the genetic purity of *S. bicolor* during foundation as well as certified seed production, but there is a tolerance for some level of impurity, and strict adherence to recommended isolation distances is considered effective (e.g., CCIA, 2019). However, the potential transfer of traits such as herbicide resistance from *S. bicolor* to *S. halepense* is expected to lead to field‐level management issues. Due to genetic similarities between *S. bicolor* and *S. halepense*, there is no herbicide that can selectively control *S. halepense* plants in an *S. bicolor* crop (Bagavathiannan et al., 2018). While herbicide‐resistant *S. bicolor* may allow for selective control of *S. halepense*, transfer of resistance to *S. halepense* through hybridization will complicate its management (e.g., Kershner, 2010). The frequency of herbicide resistance obtained through gene flow is expected to be orders of magnitude greater than resistance obtained via spontaneous mutation.

Despite the practical significance of gene flow between *S. bicolor* and *S. halepense* in agricultural systems, the factors governing gene flow are poorly understood. Previous studies establish that gene flow between *S. bicolor* and *S. halepense* can occur in either direction (i.e., *S. bicolor* as the male or female parent) (Endrizzi, 1957; Hadley, 1958). In terms of rate of hybridization in field conditions, Arriola and Ellstrand (1996) studied the rate of gene flow from *S. bicolor* to *S. halepense* and found that gene flow decreased with increasing distance, with rates as high as 2% at 100 m from the pollen source. However, there are no reports on the outcrossing rates with *S. bicolor* as the female parent under field conditions.

In a greenhouse study by Hodnett et al. (2019) of *S. bicolor* × *S. halepense*, the majority of hybrid seedlings were tetraploids. A logical mechanism for producing progeny between these two species is unreduced gametes (2*n* gametes) in *S. bicolor*. Unreduced gametes, which are widely reported among plant species (De Wet & Harlan, 1975; Kreiner et al., 2017) retain the 2*n* chromosome number of the sporophyte compared to normal gametes, which reduce to 1 *n* chromosomes. In the majority of cases, 2*n* gametes result from restitution either in the first or second division of the meiotic cycle (De Storme & Geelen, 2013). Because 2*n* gametes have been found to be heritable in alfalfa [*Medicago sativa* (L.) and in potato (*Solanum tuberosum* (L.))], breeders of these crops use them to transfer germplasm across ploidy levels (McCoy, 1982; Ortiz et al., 1991). Hodnett et al. (2019) found significant differences between two genotypes of *S. bicolor* for the frequency of tetraploid progeny from interspecific hybridization, implying that genetic factors could be affecting the frequency of 2n gamete formation.

Pollen load and competition also govern outcrossing rates between plant populations. For example, St. Amand et al. (2000) demonstrated an increase in outcrossing frequencies with increasing pollen load in alfalfa. Likewise, Ghersa et al. (1994) showed in ryegrass that outcrossing levels by an undesirable biotype could be minimized by an increased pollen load from the desirable biotype. Alternatively, male sterility in the female parent represents the extreme scenario; no self‐pollen competition. As such, male sterility in the female parent allows for the estimation of maximum hybridization potential between the two species. Scenarios of male sterility do occur in *S. bicolor* under practical field conditions. A significant portion of the sorghum grain returns to the field through harvest losses, and they are also spilled in fields and along roadsides during transportation. The commercial grain sorghum lines are typically hybrids, made using cytoplasmic male sterility in the seed parent. The grain harvested from these commercial hybrids in production fields are selfed progenies that segregate for male sterility, and approximately 25% of them will be male sterile (Ohadi et al., 2017). Such sterile plants can coexist with *S. halepense* as volunteers within the production fields or as feral plants along roadsides (Ohadi et al., 2018), greatly improving the chances of outcrossing with *S. halepense*.

To identify the genetic and environmental factors that influence crop‐wild gene flow in this system, the objectives of this study were to (1) estimate the frequency of 2n female gamete production and frequency of outcrossing with *S. halepense* as influenced by *S. bicolor* parental genetic background, and (2) elucidate the influence of pollen fertility/load in *S. bicolor* on the rates of outcrossing with *S. halepense*.

## 2 MATERIALS AND METHODS

**FIGURE 1:**
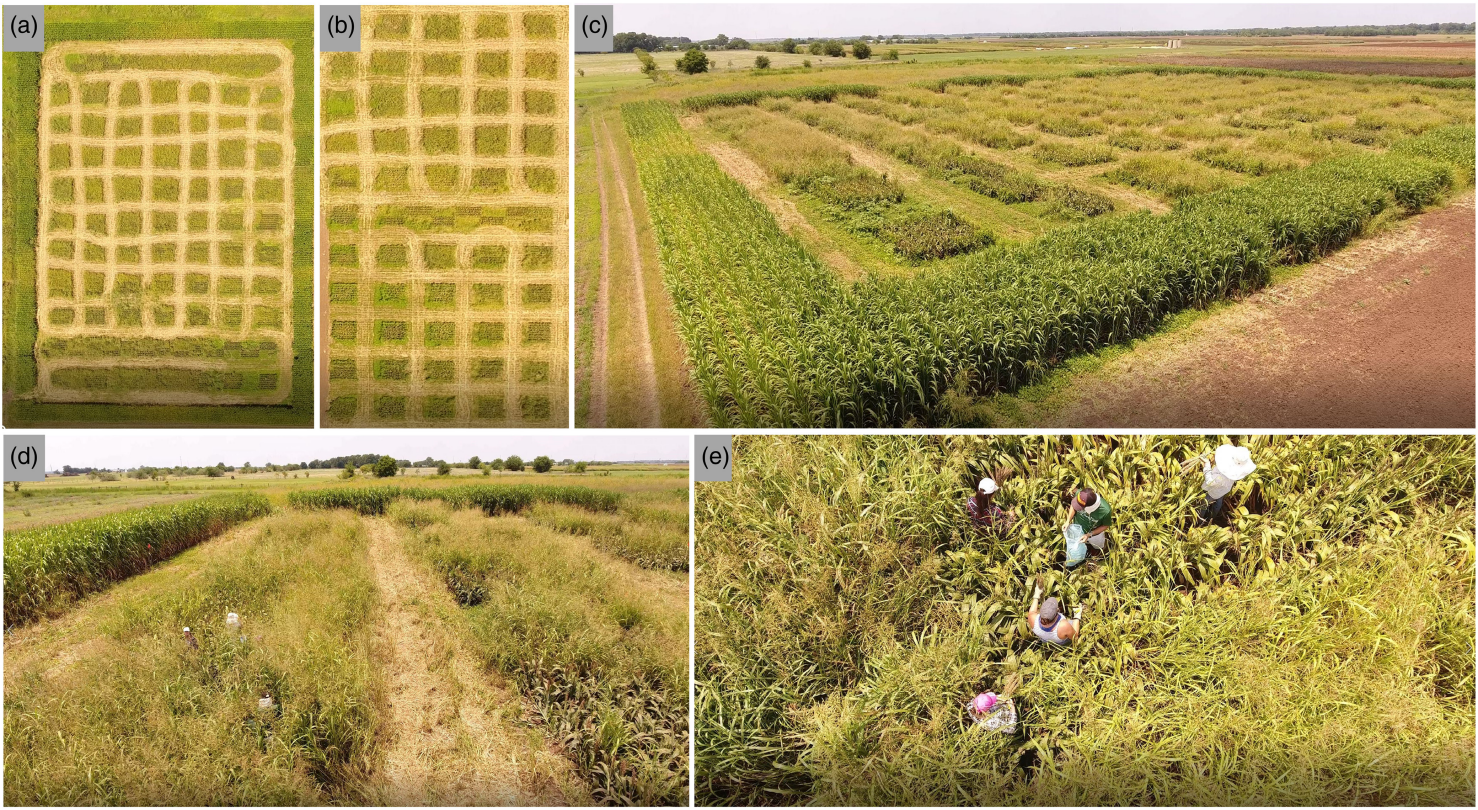
Images showing the experimental setup. (a) Aerial view of Experiment I with male sterile *Sorghum bicolor* genotypes, (b) Aerial view of Experiment II with male fertile *S. bicolor* genotypes, (c) A tall growing biomass sorghum border established around the plots in Experiment I, (d,e) View of the plots showing *S. bicolor* rows surrounded by natural *S. halepense* infestation in the experimental field.

Two field experiments (experiments *I* and *II*) were conducted in 2018 and 2019 at the Texas A&M field research facility near College Station, TX (Figure 1). The mean annual temperature for this location is 20.6°C and the average annual precipitation is 1018 mm. The aim of *experiment I* was to determine the frequency of interspecific hybridization between 12 male‐sterile *S. bicolor* seed parent lines with *S. halepense* as the sole pollinator (i.e., absence of *S. bicolor* pollen). This experiment estimated the upper limits of hybridization between the two species and established if hybridization rates vary among these seed parents. Further, the frequency of tetraploid progeny provided an estimate of 2n gamete frequencies in the female gamete of these lines. In *experiment II*, the frequency of outcrossing in 12 male‐fertile *S. bicolor* lines with *S. halepense* was evaluated. In this situation, pollen fertility in *S. bicolor* and the resulting competition between *S. bicolor* and *S. halepense* pollen was expected to mitigate interspecific hybridization. This experiment allowed for more realistic estimations of outcrossing under normal agronomic conditions. This study does not involve any human subjects, and thus informed consent is not required.

### 2.1 Experiment I

#### 2.1.1 Plant materials

The 12 male‐sterile *S. bicolor* seed parental lines (A‐lines) in this test were specifically selected because they are established seed parents for the production of elite *S. bicolor* hybrids (Table 1). Male sterility in all 12 genotypes was caused by A1 cytoplasmic male sterility (CMS) type.

**TABLE 1:** The 12 *Sorghum bicolor* parental genotypes utilized in *experiments I* and *II*.

S. No.	*S. bicolor* genotype	Pedigree	References
1	B/A.Tx2752	BTx399///BTx378//Tx378/KS30	Johnson et al. (1982)
2	B/A.Tx2921	74C5462‐1/BTx615	Rooney (2003)
3	B/A.Tx2928	RS4906/BTx399//RS4906	Rooney (2003)
4	B/A.Tx3408	Tx631/08PR047	Mbulwe et al. (2016)
5	B/A.Tx378	SA378	Stephens and Karper (1965)
6	B/A.Tx623	BTx3197/SC170‐6‐4	Miller (1977)
7	B/A.Tx626	BTx378/SC110‐6	Miller (1986a)
8	B/A.Tx631	BTx378/SC110‐9//BTx615	Miller (1986b)
9	B/A.Tx642	IS12555/Tx436//IS12555	Rooney (2003)
10	B/A.Tx645	BTx623//BTx625/BTx642	Rooney (2003)
11	B/A.TxArg‐1	MR807/BTx624	Miller et al. (1992)
12	B/A.Tx3447	BTx643/BTx635	Rosenow et al. (2021)

*Note*: The B‐line version is male fertile while the A‐line version is male sterile using A1 cytoplasm.

#### 2.1.2 Experimental setup and field maintenance

**FIGURE 2:**
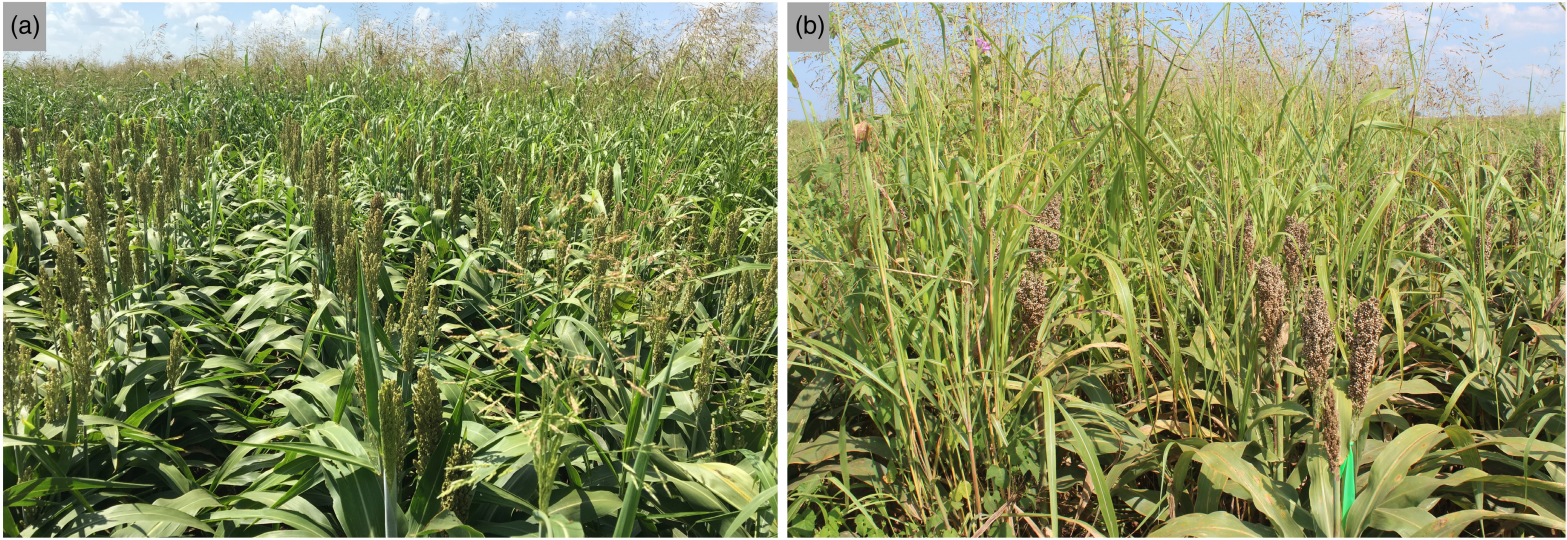
Natural infestation of *Sorghum halepense* in the experimental site where (a) male sterile, and (b) male fertile *Sorghum bicolor* genotypes were established.

The experimental units were laid out in a randomized complete block design with four replications. Each plot was eight rows wide (0.76 m row spacing) and 6.7 m long. The *S. bicolor* genotypes were planted into a field uniformly infested with *S. halepense*, on April 20 in both 2018 and 2019. The natural *S. halepense* infestation served as the pollinator parent (Figures 1 and 2). To eliminate any extraneous sources of *S. bicolor* pollen, the tall bioenergy sorghum hybrid ES5200 was planted around the experimental site as a buffer (Figure 1). ES5200 is photoperiod‐sensitive energy sorghum that has dense growth and does not flower in Central Texas, meaning it did not shed any pollen during the life of the study.

A pre‐emergence application of pendimethalin (Prowl H_2_O^®^) was made at 2130 g ai/ha immediately after planting of *S. bicolor*. An early‐postemergence application of *S*‐metolachlor+ atrazine (Bicep II Magnum®) at a rate of 1736 + 2242 g ai/ha and a mid‐postemergence application of a premix of 79 g ai/ha pyrasulfotole and 445 g ai/ha bromoxynil (Huskie®) were applied for additional weed control. These applications were made mainly to control morning‐glories (*Ipomoea* spp.) and other broadleaved weeds, and had little activity on *S. halepense*. Insecticide [Prevathon® (56.5 g ai/ha), Silencer® (17.5 g ai/ha), and Sivanto® (153.6 g ai/ha)] applications were made to control sorghum midge, sugarcane aphid, armyworm, and other insect pests. Further, a premix of propiconazole 127.6 g ai/ha and 76 g ai/ha azoxystrobin (Quilt®) was applied to pre‐emptively control ergot infestations during seed development.

#### 2.1.3 Data collection

Plots were monitored daily during *S. bicolor* anthesis to scout and remove any sources of *S. bicolor* pollen through potential fertility revertants in the plots. These revertants are known to occur in CMS male‐sterile *S. bicolor* at low frequencies (<1%). The *S. bicolor* panicles in each plot were also scored for successful fertilization at the early seed development stage to verify the presence of sufficient *S. halepense* pollen in the experimental field area. At maturity or at the post‐hard dough stage (~35 days after flowering), at least 50 mature panicles were randomly selected and manually harvested from the center of each plot. Given that the vast majority of panicles had a low seed set (<3%), any panicles with a full seed set were likely male fertile revertants that were not rogued; they were excluded from the samples along with adjacent panicles.

The harvested panicles were stored in mesh bags and air‐dried at room temperature. Aluminum phosphide (Weevil‐cide®) was applied at a rate of 145 tablets per 28.31 m^3^ (9.2 g ai/m^3^) to control moths and other pests during seed storage. Each panicle was individually threshed using an Almaco BT14 Belt thresher. Threshed seeds were cleaned using an aspirator to remove chaff and other foreign material. The total number of mature seeds produced in each panicle was determined using a seed counter. Total number of florets produced by each sterile panicle was estimated each year, based on the maximum plot average seed set values obtained from the male‐fertile version of each *S. bicolor* line (50 panicles/line) in *experiment II* each year.

#### 2.1.4 Quantification of outcrossing

To quantify the outcrossing frequency, true hybrids in the harvested F_1_ seed were confirmed based on ploidy level. Ploidy of all F_1_ progeny was determined through a flow cytometry assay (see below), using an ACURI C6 flow cytometer (Becton Dickinson and Co., Franklin Lakes, NJ). Any progeny identified as diploid (2n = 2× = 20) were the result of either self‐ or cross‐pollination with *S. bicolor* pollen (due to either sterility break or external sources). For additional confirmation, all diploid seedlings from the flow cytometry assay were transplanted to the field to verify the *S. bicolor* phenotype. Any progeny with triploid or higher ploidy levels were considered interspecific hybrids.

For each genotype, a sub‐sample (3 g, about 150 total seeds) of the F_1_ putative hybrid seed was drawn. Half of that seed was utilized for flow cytometry analysis to determine ploidy, and the rest was planted in the field for evaluating field emergence and establishment. Germination of seed in Petri dishes and seedling establishment in the greenhouse for flow cytometry analysis also provided data on seedling establishment potential under ideal conditions.

### 2.2 Flow cytometry for ploidy determination

Ploidy of the F_1_ progeny was determined through flow cytometry using an ACURI C6 flow cytometer (Becton Dickinson and Co., Franklin Lakes, NJ). Seed were treated with a fungicide mix containing 5 mL MaximXL® (fludioxonil and mefenoxam) and 19 mL ApronXL® (mefenoxam) in 1 L of water and placed on filter paper (Whatman no. 1) in Petri dishes. Seedlings at the 1‐leaf stage were transferred to 50‐cell plastic trays filled with potting soil (LC1 potting soil mix, Sungro Horticulture, Canada) and placed in a controlled environment greenhouse maintained at 32/28°C day/night temperature and a 14‐h photoperiod.

When seedlings reached about 15‐cm in height, a small piece of the newest leaf tissue (approx. 1 cm^2^) was harvested and chopped with a single‐edged razor blade in cold, modified woody plant nuclei isolation buffer (WPB) (Loureiro et al., 2007). WPB is an aqueous solution consisting of 20 mM tris (hydroxymethyl)aminomethane (C_4_H_11_NO_3_), 4 mM magnesium chloride 6‐hydrate (MgCl_2_ 6H_2_O), 2 mM ethylenedinitrilo‐tetraacetic acid ([HO_2_CCH_2_]2N[CH_2_]2N[CH_2_CO_2_‐Na]2H_2_O), 86 mM sodium chloride (NaCl), 10 mM sodium metabisulfite (Na_2_SO_3_), 1% polyvinylpyrrolidone (PVP‐12310), and 0.5% (v/v) Triton X‐100 at pH 7.5. Further, RNase A (PureLink™, Invitrogen, Carlsbad, CA) was added to WPB at 5 mg L^−1^ just prior to use. The slurry of each plant sample was filtered through a 30 μm CellTrics disposable filter (Partec, Munster, Germany) and the nuclei in the filtered buffer were stained with 50 μg mL^−1^ propidium iodide (Sigma‐Aldrich, St. Louis, MO). Samples were placed on ice until they were analyzed. The propidium iodide‐labeled nuclei in each sample (at least 200 nuclei) were analyzed for ploidy in the flow cytometer with an air‐cooled laser operating at 488 nm; the fluorescence collected through a 585/20 band pass filter (Becton Dickinson and Co., Franklin Lakes, NJ) was quantified (CV < 10%). The nuclei of a sorghum‐sugarcane hybrid with known ploidy were used as a standard for comparison during the evaluation, and the ploidy level was determined by the ratio of their respective G1 peaks.

### 2.3 Experiment II

#### 2.3.1 Plant materials

In this evaluation, the male‐fertile versions (B‐lines) of the 12 parental lines evaluated in *experiment I* were used (Table 1).

#### 2.3.2 Experimental setup and field maintenance

In both years, the study was conducted in a randomized complete block design, with 4 replications, for a total of 48 plots. Plot sizes, planting dates, and general field establishment and maintenance were identical to those described in e*xperiment I*. While the test was planted in a field with a dense infestation of *S. halepense*, a no‐sorghum buffer equivalent to the plot size was maintained around each plot to provide sufficient *S. halepense* pollen. This field was adjacent to *experiment I*, but a biomass sorghum border was not required.

#### 2.3.3 Data collection

Field data collection was identical to that of *experiment I* until threshing and seed counting. In this experiment, the total number of florets produced by each panicle was estimated based on the seed set value obtained from each plot (50 panicles/plot).

#### 2.3.4 Quantification of outcrossing

**FIGURE 3:**
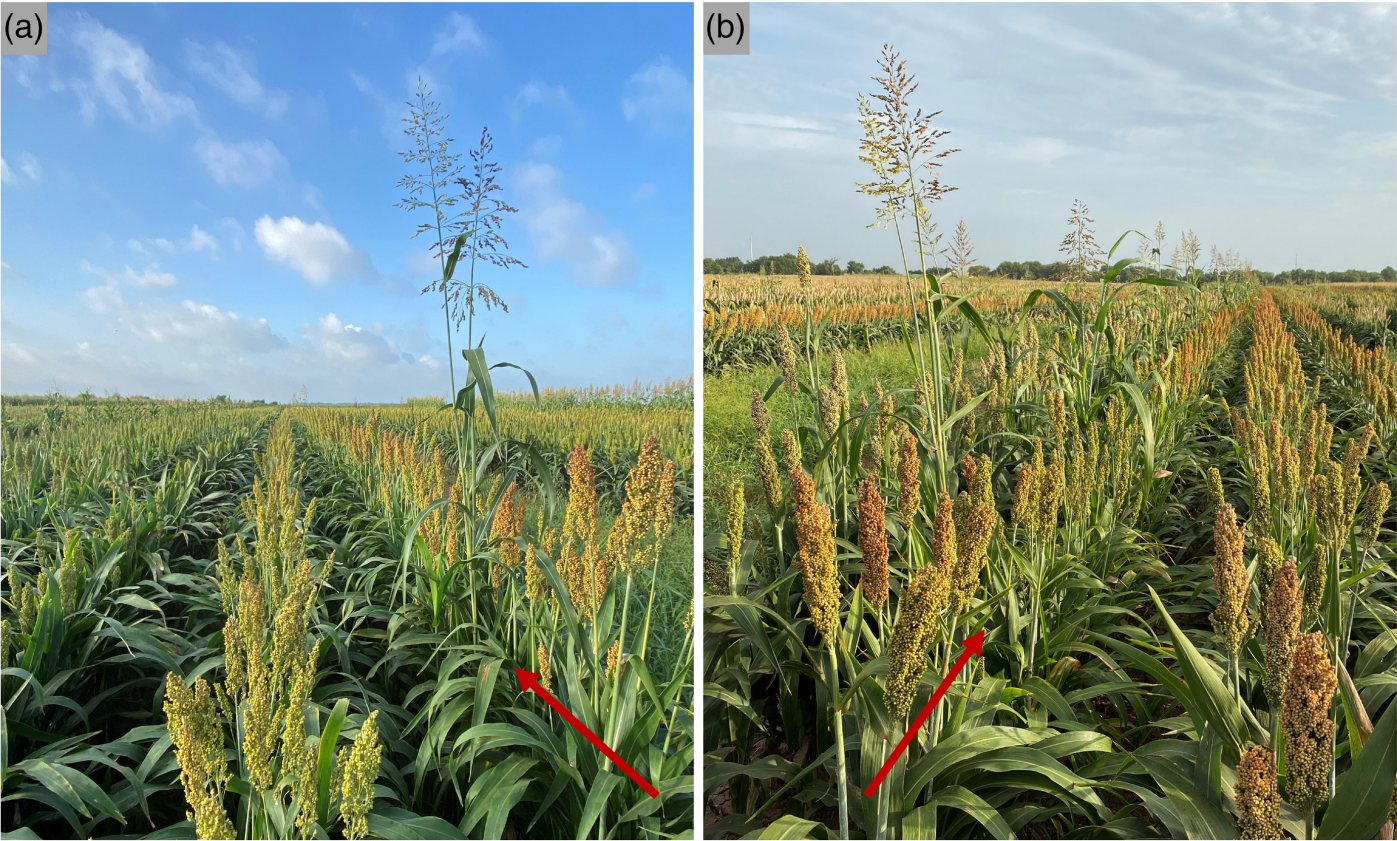
Image reflecting the phenotype of F_1_ interspecific hybrids (tall plants), compared to regular diploid *Sorghum bicolor* plants surrounding it, in the F_1_ progeny rows established for the tested genotypes. Hybrids are taller, with intermediate leaf width, and open panicles that neither resemble that of *Sorghum halepense* nor *S. bicolor*. (a) F_1_ hybrid frequency in an *S. bicolor* genotype with low outcrossing potential (Tx2752), compared to (b) a genotype with high outcrossing potential (Tx626).

The 50 panicles from each plot were threshed, the seed was bulked among the panicles, and a 300 g sample (~30,000 seeds) was field planted in progeny rows (row spacing: 76 cm) on April 18, 2019, and April 13, 2020. Thus, approximately 120,000 F_1_ progeny were screened for each *S. bicolor* genotype across the four field replications. Prior to planting, the seed was treated with Concep®, Apron XL®, and Cruiser®. At mid‐anthesis, the rows were visually screened and every potential interspecific hybrid (i.e., off‐type) within the row was identified by their phenotype (Figure 3). Ploidy of the off‐types was determined using flow cytometric analysis as previously described. Additionally, the actual number of all established plants in the progeny rows (among the ~30,000 seeds planted for each plot) was counted for calculating outcrossing frequency.

### 2.4 Statistical analysis

#### 2.4.1 Calculation of 2n gamete and outcrossing frequency

##### Experiment I

The frequency of 2n gametes was calculated for each genotype using the following formula:

$$\begin{array}{cc} \text{2n gamete frequency}= & [\mathrm{sum}\;\text{of tetraploids}+\text{hexaploids}\;\mathrm{per}\;\text{panicle}/\\ & \text{total number of florets}\;\mathrm{per}\;\text{panicle}]*100 \end{array}$$

and the outcrossing frequency was calculated for each genotype (at an individual panicle level) using the following formula:

$$\begin{array}{cc} \text{Outcrossing frequency}= & (\text{number of hybrids produced}\;\mathrm{per}\;\text{panicle}/\\ & \mathrm{avg}\;\text{number of total florets}\;\mathrm{per}\;\text{panicle})*100 \end{array}$$

where the total florets per panicle were estimated based on maximum plot average seed set for the fertile version of the same parental line in *Experiment II*.

##### Experiment II

The outcrossing frequency was calculated for each genotype (at an individual plot level) using the following formula:

$$\begin{array}{cc} \text{Outcrossing frequency}= & (\text{number of hybrids produced}\;\mathrm{per}\;\text{plot}/\text{total number of}\\ & \text{plants established in progeny rows for the plot})*100 \end{array}$$

#### 2.4.2 Statistical model and means comparison

For both *experiments I* and *II*, the frequency of 2n gamete production and outcrossing rate as influenced by different *S. bicolor* parental backgrounds and pollen load was analyzed using the GLIMMIX procedure of the Statistical Analysis Software (SAS Institute Inc., Cary, NC). The GLIMMIX procedure was used because the evaluation of the data revealed a non‐normal distribution and GLIMMIX is a robust procedure that is able to handle non‐normal data in experiments with multiple environments. Prior to GLIMMIX, data transformations were considered and explored, but no transformation was sufficient to normalize the data. A link function (link = log) was used in the GLIMMIX model statement to account for this. The statistical model for analysis included genotype_i_, year_k_, replication, genotype*year, and error. Genotype and year were considered as fixed effects, and replication (nested within years) as the random effect. Because this analysis revealed a statistically significant and meaningful genotype*year interaction, the 2 years were analyzed separately. Following ANOVA, mean separations were carried out using the Fisher's protected Least Significant Difference (LSD) method (α = 0.05). Further, a t‐test was also conducted to compare F_1_ hybrid establishment data between greenhouse and field environments, using PROC TTEST in SAS.

## 3 RESULTS

The infestation of *S. halepense* was uniform across the plots and exhibited good flowering synchrony with *S. bicolor* (Figures 1 and 2). High fertilization scores in the male‐sterile lines across the experimental field revealed that enough *S. halepense* pollen was available in the area (data not presented). In most plots, fertilization was evident approximately 7 days post‐anthesis as small seeds began to appear between the glumes of each floret. However, between 15 and 20 days post‐anthesis, the vast majority of seeds collapsed, indicating that endosperm development had failed and the embryo had died. Thus, there was a high degree of acceptance of *S. halepense* pollen by *S. bicolor*, but endosperm failure resulted in most of the seed being lost. Since the majority of the *S. bicolor* gametes would be reduced (n = x = 10), fertilization with *S. halepense* would produce large numbers of triploids; therefore, the collapsing seed was likely triploid. Endosperm failure resulting in seed collapse in *S. bicolor* has been observed previously (Hodnett et al., 2019; McClure, 1965), with the phenomenon given the term “triploid block” (Marks, 1966). However, the surviving hybrids that did emerge were phenotypically distinct from both *S. bicolor* and *S. halepense*. The hybrids were much taller (>2 m height) compared to the diploid *S. bicolor* seed parents with open panicles and larger florets than that of *S. halepense* (Figure 3). Their leaves were typically wider, and the culms were thicker than that of *S. halepense*, but not as wide or thick as leaves and stems in regular *S. bicolor* (personal observations, data not shown). Prior reports and observations of the hybrid phenotype obtained with controlled crosses also documented similar characteristics (Dweikat, 2005; Hadley, 1958).

### 3.1 Ploidy status of the interspecific hybrids

True interspecific hybrids were produced in most seed parent lines, although the numbers varied across parents and years. The distribution of different ploidy types in the F_1_ progenies was relatively consistent across genotypes and years. By far, the most commonly observed ploidy level was tetraploid in both experiments (Tables 3, 4).

### 3.2 Outcrossing in male‐sterile genotypes

**FIGURE 4:**
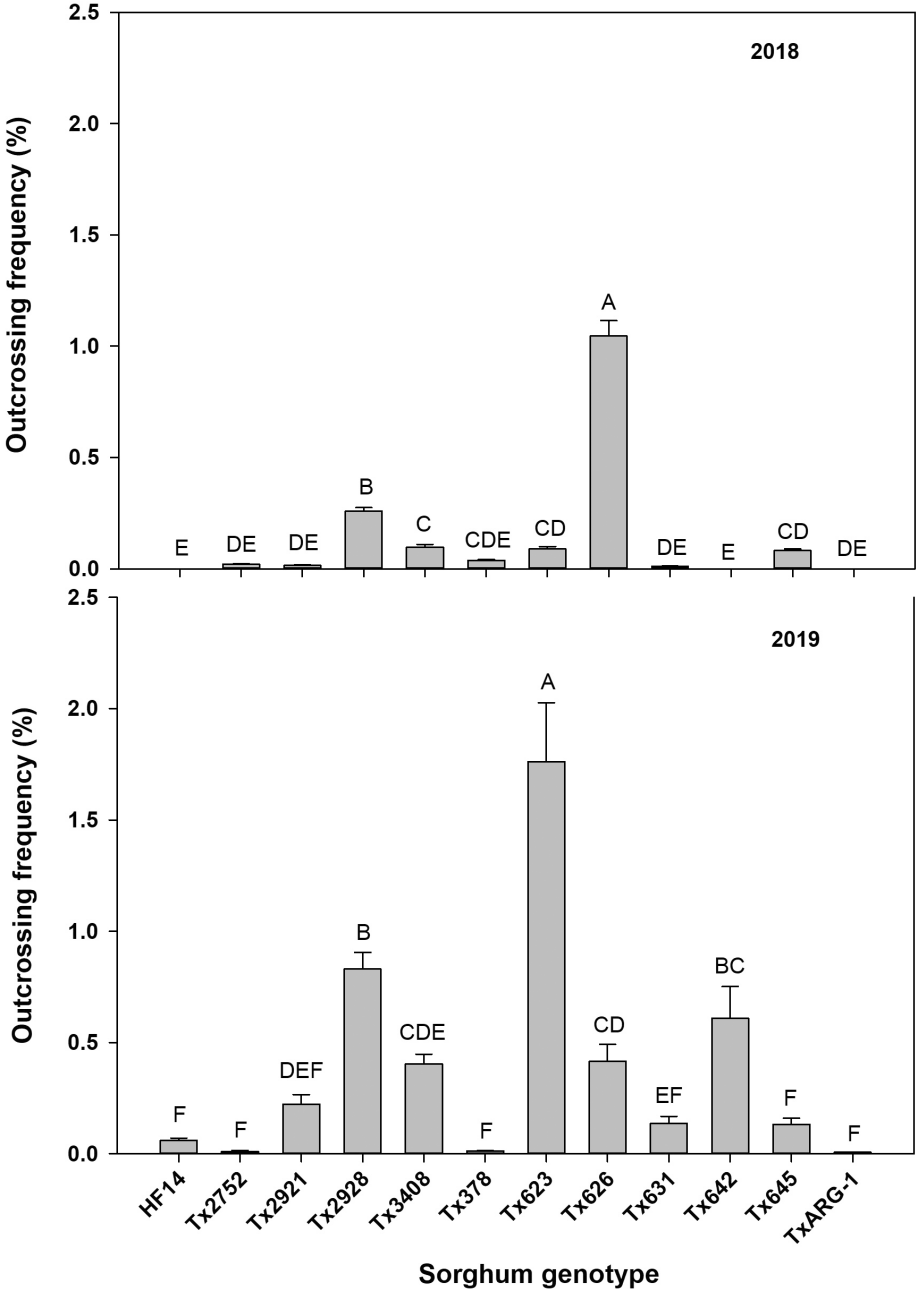
Outcrossing frequencies in 12 different male sterile (A1 cytoplasmic male sterility) *Sorghum bicolor* parental lines only in the presence of *Sorghum halepense* pollen evaluated in 2018 (top) and 2019 (bottom) near College Station, Texas. The letters above the bars indicate significant differences at α = 0.05.

Data analysis revealed significant effects for both genotype (df = 11) and year (df = 1) as well as genotype*year interaction (df = 11), for both 2n gamete frequency and outcrossing rate (Table 2). We failed to detect any treatment*replication interactions and due to high volume of samples to be tested, only two of the four field replications were utilized for quantification of outcrossing. In 2018, outcrossing frequency ranged from 0.005% to 1.045% (average: 0.143%) across the 12 A‐lines (i.e., male‐sterile *S. bicolor* lines) while in 2019 outcrossing ranged from 0.007% to 1.762% (average: 0.379%) (Figure 4). Of the genotypes evaluated, ATx626 had the highest outcrossing at 1.045% in 2018; ATx623 was the highest in 2019 (1.762%) (Figure 4). The shift in response across years for these two lines is one reason why the interaction term was significant. However, it should be noted that some genotypes consistently produced low and high levels of interspecific hybridization across years; this indicates that some A‐lines are consistent in their tendency to produce greater frequencies of 2n gametes (Figure 4).

**TABLE 2:** The estimated 2n gamete frequency was observed in 12 male sterile A‐line *Sorghum bicolor* seed parents sterilized in A1 cytoplasm and pollinated exclusively by *S. halepense* in 2018 and 2019.

*S. bicolor* genotype	2n gamete frequency (%)^a^			
	2018		2019	
Tx626	1.026	A	0.415	CD
Tx2928	0.267	B	0.802	B
Tx623	0.092	C	1.762	A
Tx645	0.085	C	0.055	E
Tx3408	0.057	CD	0.252	CDE
Tx378	0.049	CD	0.004	E
Tx2921	0.019	CD	0.191	DE
Tx2752	0.013	D	0.000	E
Tx631	0.011	D	0.119	E
TxARG‐1	0.007	D	0.006	E
Tx642	0.005	D	0.514	C
HF14	0.004	D	0.029	E

*Note*: The 2n gamete frequency was determined by dividing total florets in a panicle by the number of tetraploid and hexaploid progeny produced from that panicle.

^a^ Means separated by letters within a column were significantly different at α = 0.05.

**TABLE 3:** Distribution of interspecific hybrids derived from 12 male sterile *Sorghum bicolor* parental lines that were pollinated with *Sorghum halepense* in College Station in 2018 and 2019.

*S. bicolor* genotype	2018		2019	
	Triploid	Tetraploid	Triploid	Tetraploid
A.Tx3447	0	0	0	1
A.Tx2752	1	4	2	0
A.Tx2921	0	6	1	16
A.Tx2928	0	11	1	24
A.Tx3408	1	2	2	3
A.Tx378	0	9	4	1
A.Tx623	0	15	0	57
A.Tx626	0	33	0	15
A.Tx631	1	1	4	1
A.Tx642	0	0	6	28
A.Tx645	0	20	1	9
A.TxArg‐1	0	0	1	2
Total numbers	3	101	22	157
Percentage	2.9	97.1	12.3	87.7

**TABLE 4:** Distribution of different ploidy types in the F_1_ progeny derived from male fertile *Sorghum bicolor* parental lines pollinated with *Sorghum halepense* in College Station in 2018 and 2019

	2018		2019			
Parental line	Triploid	Tetraploid	Triploid	Tetraploid	Pentaploid	Hexaploid
B.Tx3447	0	0	5	1	0	0
B.Tx2752	0	0	5	0	0	0
B.Tx3408	0	0	1	2	0	0
B.Tx378	0	0	4	1	0	0
B.TxArg‐1	0	0	1	2	0	0
B.Tx2921	3	1	0	0	0	0
B.Tx2928	0	2	1	9	0	0
B.Tx623	0	1	5	68	1	0
B.Tx626	0	11	7	172	0	1
B.Tx631	0	1	5	1	0	0
B.Tx642	2	0	5	2	0	0
B.Tx645	0	0	20	20	0	0
Total numbers	5	16	59	278	1	1
Percentage	23.8	76.2	17.4	82.0	0.3	0.3

### 3.3 Outcrossing in male fertile genotypes

**FIGURE 5:**
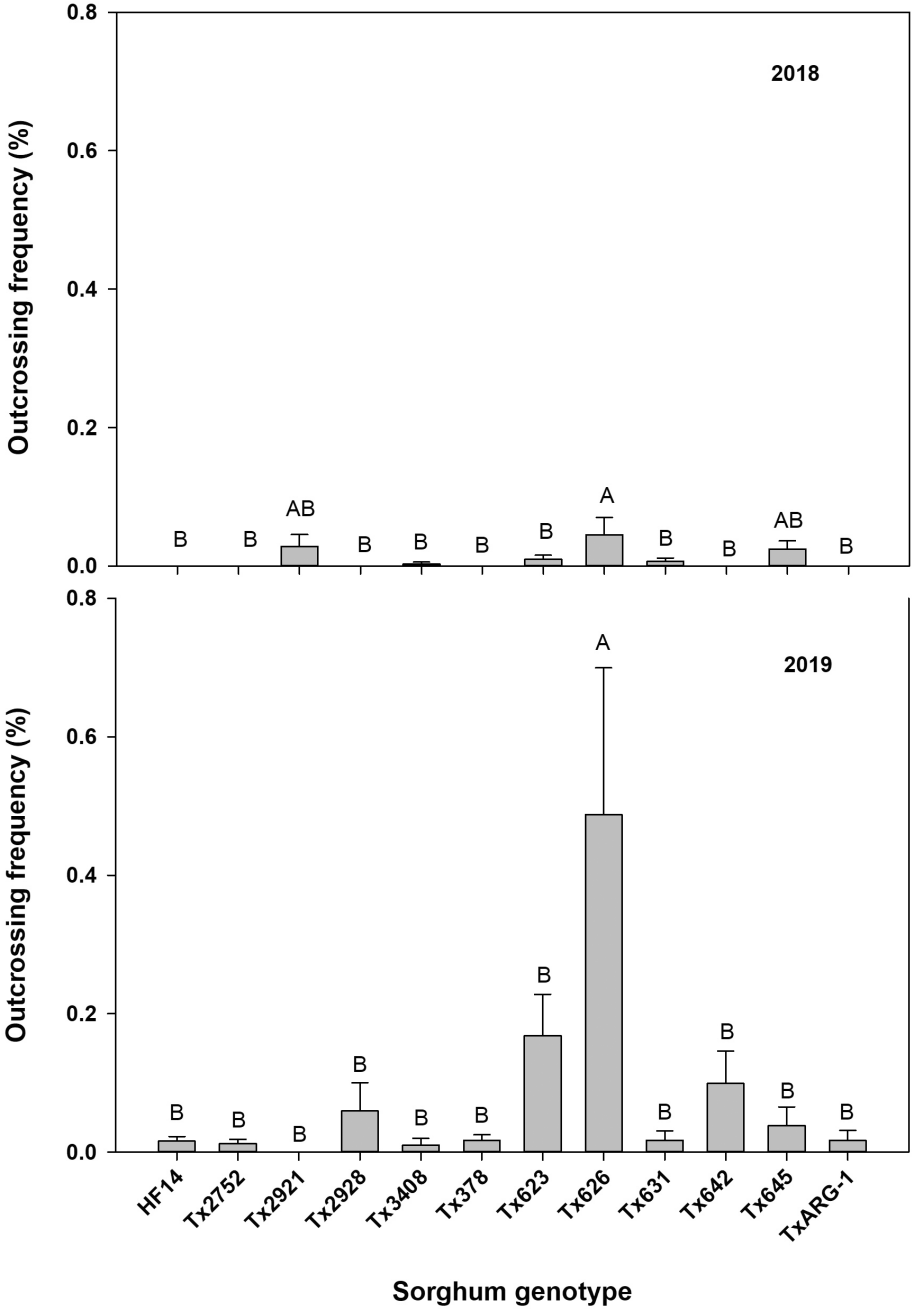
Outcrossing frequencies occurring in 12 different male fertile *Sorghum bicolor* parental lines in the presence of both *S. bicolor* and *Sorghum halepense* pollen were evaluated in 2018 near College Station, Texas. The letters above bars indicate significant differences at α = 0.05.

In the B‐lines, the outcrossing frequencies were orders of magnitude lower than in the male sterile lines (Figure 5). This trend was consistent in both years, although the outcrossing rates were slightly higher in 2019. In both 2018 and 2019, the highest outcrossing frequency was reported in Tx626 at 0.045% and 0.49%, respectively; most other genotypes had significantly lower levels (Figure 5). Self‐fertility in these B‐lines clearly reduces pollination and fertilization by *S. halepense* pollen, but it does not exclude it. Differences among years indicate that the environment also influences interspecific hybridization rates. Overall, rates of outcrossing among the different A and B‐lines of *S. bicolor* followed similar trends within each group, even though they were orders of magnitude lower in the latter (Figures 4 and 5). It should be noted that the outcrossing rates estimated here with the male fertile *S. bicolor* lines already account for any loss during field establishment (e.g., lack of fitness) because these were direct field grow‐outs.

### 3.4 2n gamete frequencies

The A‐lines provided a simple and efficient way to screen for 2n female gamete production in *S. bicolor* as the frequency of 2n gamete production is equivalent to the total of all tetraploid and hexaploid progenies produced. Based on that method of estimation, the frequency of 2n female gamete formation in *S. bicolor* was high in ATx626 and ATx623, and it was consistently lower in ATxArg‐1, ATx3447, and ATx2752 (Table 2).

## 4 DISCUSSION

In this research, outcrossing frequencies were determined for the presence or absence of self‐pollen competition in *S. bicolor*, which allowed for the estimation of outcrossing frequencies in both a normal and a worst‐case scenario. To our knowledge, this is the first detailed study where such a range of *S. bicolor* genetic backgrounds was compared under natural field conditions.

The majority of the progeny produced in the A‐lines (i.e., male‐sterile *S. bicolor*) were interspecific hybrids. These progeny phenotypically exhibited the characteristics expected of an interspecific hybrid between *S. bicolor* and *S. halepense*. These characteristics include tall growth (>2 m in height), with thicker stems and wider leaves than that of *S. halepense*, but thinner and narrower than that of *S. bicolor* (Figure 3). These phenotypic characteristics are important as they allow for positive identification of *S. bicolor* × *S. halepense* hybrid progenies under field conditions. The hybrid progenies were predominantly tetraploid, but triploids manifested at low rates. Most of the hybrids were male sterile, but it is difficult to draw any inferences from this information because F_1_ progeny from such hybridization could be sterile due to A1 CMS cytoplasm in the female parent, cytological incompatibilities, or other unknown factors. Adugna and Bekele (2013) observed that crosses of the crop (female)‐wild (male) *S. bicolor* were fertile, which indicates that the fertility status of the F_1_ hybrids cannot be generalized and that fertility of any hybrid must be confirmed.

Diploids observed in the progeny were likely the result of *S. bicolor* pollen in the experimental area from either outside sources or sterility breaks that can occur even at individual floret levels (Papathanasiou & Lessman, 1969). Field observations of a sub‐sample of the diploid progeny showed a *S. bicolor* phenotype and thus, the diploid progeny data were excluded from the calculation of outcrossing frequency.

Outcrossing frequencies ranged from 0 to 0.49% in the B‐lines (i.e., male fertile *S. bicolor*) and between 0 and 1.762% in the A‐lines. These results have two implications. First, some *S. bicolor* genotypes are clearly less or more prone to hybridize with *S. halepense*. Second, pollen load in *S. bicolor* influences hybridization with *S. halepense*; outcrossing frequencies in the absence of *S. bicolor* pollen were between one and two orders of magnitude higher than when *S. bicolor* pollen was present. Arriola and Ellstrand (1996) documented *S. halepense* × *S. bicolor* hybridization to occur at 11% frequency at the closest distance of 0.5 m and as high as 2% at 100 m from the *S. bicolor* parent, which was used as the pollinator. Though these levels of outcrossing were much higher than what was reported in the current study under conditions of male fertility in the female parent, the authors have used *S. halepense* as the female parent (as opposed to *S. bicolor* in the current study), which might have contributed to the differences observed. Evaluation of the male‐sterile genotypes pollinated only with *S. halepense* provides direct insight into the factors causing interspecific hybridization between *S. bicolor* and *S. halepense*. In this study, significant variation for outcrossing frequency was caused by both main effects (genotype and year). While environmental conditions influenced outcrossing rates, basic environmental data from the 2 years did not show obvious reasons for the difference (data not shown). Hanson et al. (2005) showed in wheat that several environmental factors, including relative humidity, rainfall, air temperature, and light intensity affected the ability of pollen to cross‐fertilize.

**FIGURE 6:**
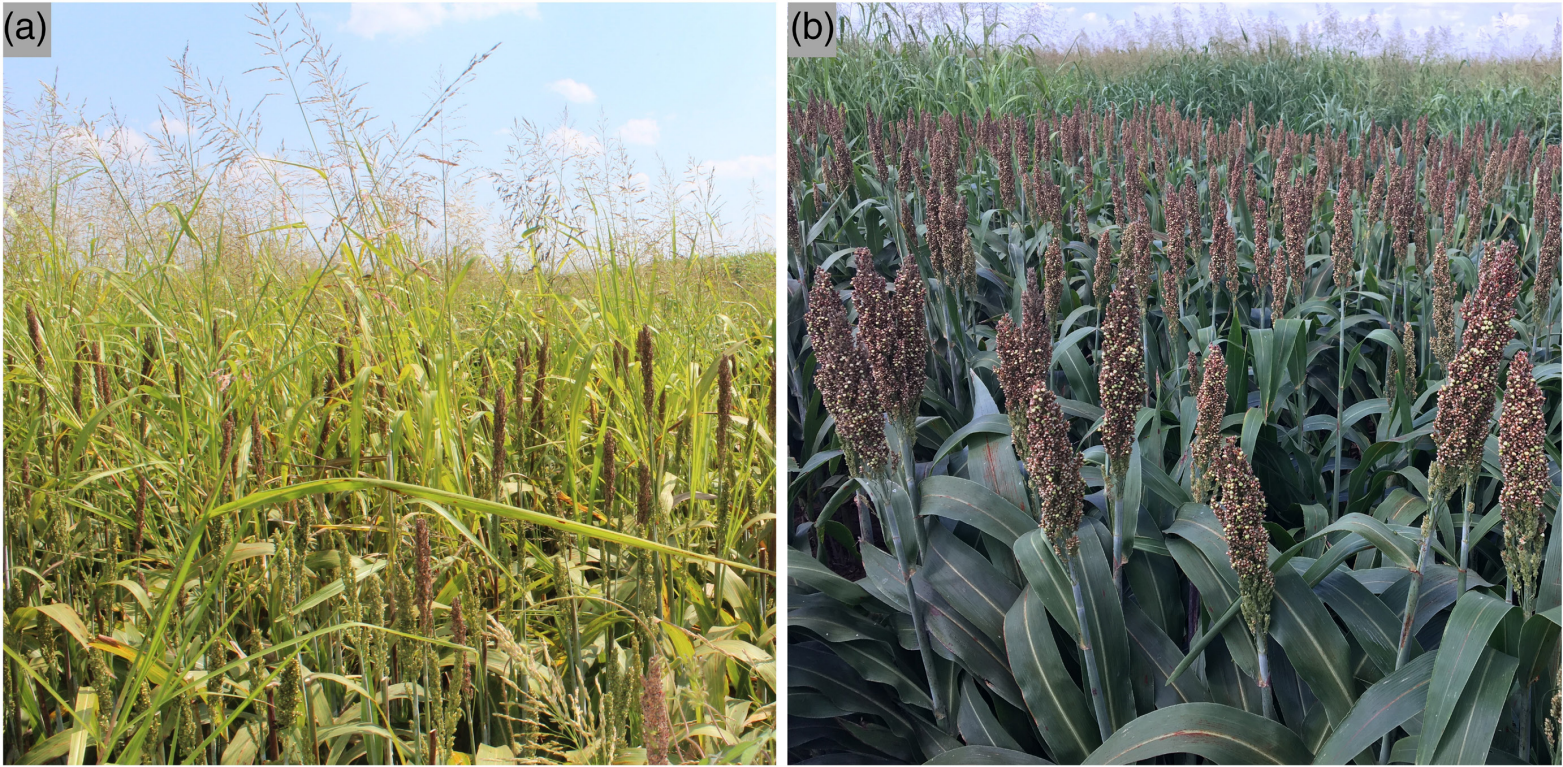
Differences in seed set between *Sorghum bicolor* genotypes with (a) low (Tx2752) and (b) high (Tx626) interspecific hybridization potential with *Sorghum halepense*.

While interactions caused some shifts in magnitude, some genotypes were consistently more likely to outcross (Tx2928, Tx626, and Tx623), while some others were consistently less likely to do so (Tx3447, Tx2752, and TxArg‐1) (Figure 6). The differences between these parental lines imply a genetic effect. A myriad of factors could cause these differences, ranging from floral structure, duration of stigma receptivity, ovule viability, and the frequency of unreduced or 2n gametes (Cisneros‐López et al., 2012; Moran et al., 2003; Nguyen et al., 2013). For instance, Tx623 consistently had high outcrossing frequencies, and it is known to have a longer duration of stigma receptivity (Moran et al., 2003).

**FIGURE 7:**
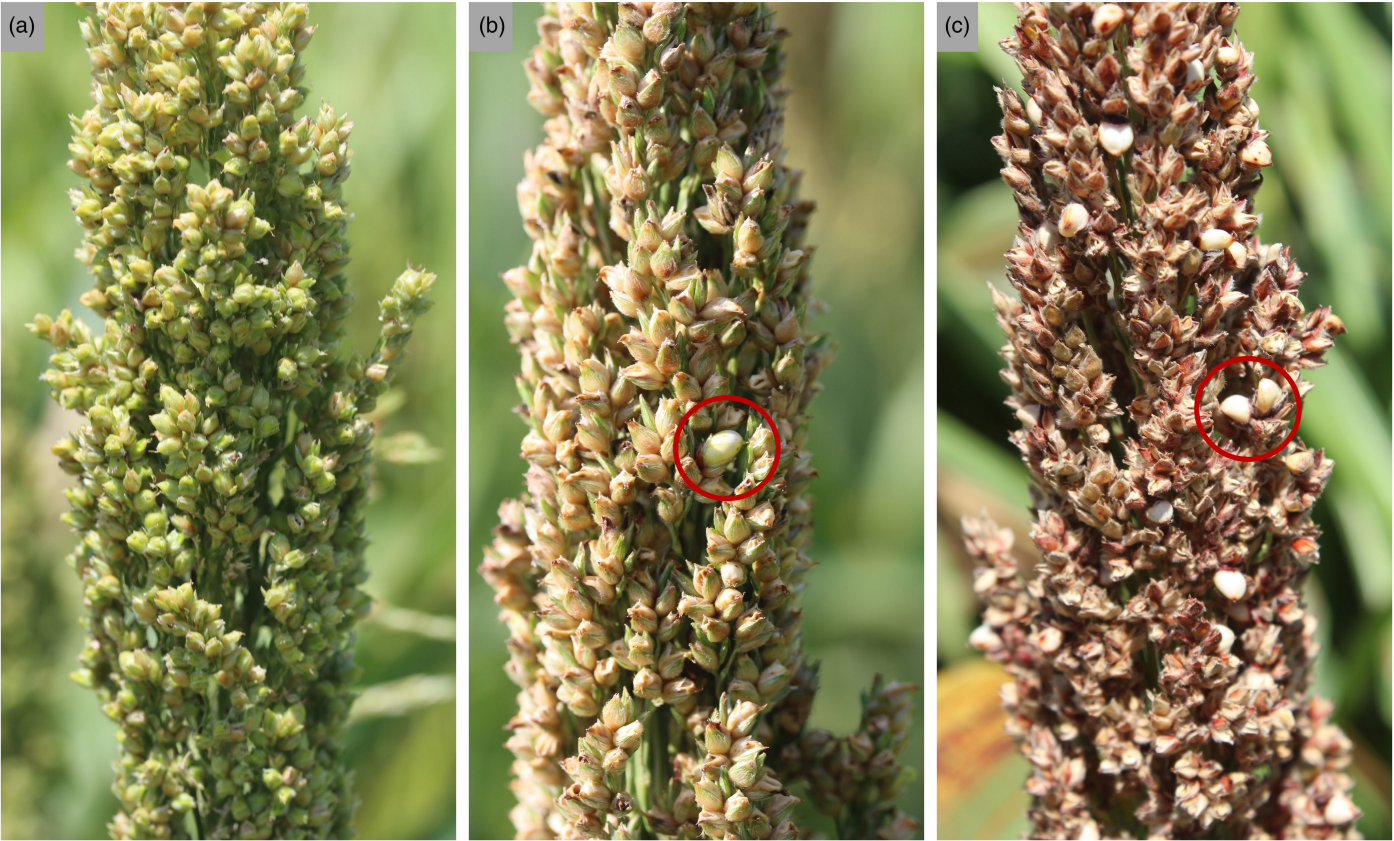
(a) Normal development of seed in *Sorghum bicolor* panicle following pollination by *Sorghum halepense*, (b) endosperm collapse in the majority of the seed, leading to fewer seed continuing to develop normally (e.g., seed highlighted in the circle), and (c) an example of the *S. bicolor* panicle with few fully developed seed at maturity.

As noted earlier, most *S. bicolor* florets in the male‐sterile lines appeared to have been fertilized by *S. halepense* pollen. Given that a triploid progeny would be expected from the union of a normal n = × = 10 gamete from *S. bicolor* and a normal n = 2× = 20 gamete from *S. halepense*, most of the florets would form a triploid progeny. However, the majority of the triploid progeny collapsed due to the failure of the endosperm (Figure 7). Endosperm failure is common in triploid progeny resulting from crosses involving unbalanced ploidies (Cox et al., 2018, Hodnett et al., 2019, Marks, 1966, McClure, 1965).

The majority of the progeny that survives are tetraploids that are formed when a 2n gamete (2n = 2× = 20) in the *S. bicolor* female forms a zygote with a normal n = 2× = 20 gamete from *S. halepense*. Because chromosome number and ploidy are balanced in this case, the endosperm is less likely to fail and these seed develop to maturity to produce viable seedlings. The 2n gamete formation has been observed in other genera such as *Lilium* (van Tuyl et al., 1989), *Lotus* (Negri & Lemmi, 1998), *Citrus* (Xie et al., 2019), *Avena* (Nikoloudakis et al., 2018), *Populus* (Zhao et al., 2017), to name a few. In fact, 2n gamete formation has been used in breeding for crops such as potato and alfalfa (De Maine, 1982; Veronesi et al., 1986), and there are even approaches to investigate the potential to stimulate 2n gamete formation (Younis et al., 2014). There are currently efforts to further discover how to control and more efficiently use these 2n gametes, but the current study was focused on the natural formation of 2n gamete as influenced by genotypic backgrounds. The preponderance of tetraploid progeny indicates that 2n gamete production is the critical factor in the production of interspecific hybrids between *S. bicolor* and *S. halepense*. These observations corroborate the findings of Hodnett et al. (2019) and indicate that hybridization frequency is related to the frequency of 2n gamete formation in different *S. bicolor* parental lines.

The results imply an underlying genetic control of 2n gamete formation in *S. bicolor*, and genetic control of 2n gamete production is well documented in other crop species. Fakhri et al. (2016) found that the frequency of unreduced gamete production was genetically controlled in hybrids produced by *Triticum aestivum* × *Aegilops triuncialis*. In an analysis of self, F_1_, F_2_, and backcross families of diploid alfalfa, McCoy (1982) determined that 2n pollen formation was controlled by a single recessive gene. In potatoes, 2n pollen and 2n eggs are each controlled by single recessive genes *ps* (Watanabe & Peloquin, 1988) and *os* (Werner & Peloquin, 1990). These results imply that genotypes could be chosen for a reduction in 2n gamete production, which would in turn reduce the frequency of interspecific hybridization. If further research assessing 2n gamete formation in *S. bicolor* reveals a heritable trait, then breeders could actively select against their formation as well, as utilized previously in potato and alfalfa (De Maine, 1982, Veronesi et al., 1986).

### 4.1 Agronomic and ecological implications

Our results confirm that *S. bicolor* × *S. halepense* hybridization does routinely occur under natural field conditions. The agronomic and ecological consequences of *S. bicolor* × *S. halepense* hybridization can be significant. The hybrids may be more weedy and problematic than the wild parent in the production fields, though this theory is yet to be tested in the *S. bicolor* × *S. halepense* hybrids (with *S. bicolor* as the female parent). The transfer of an adaptive trait (transgenic or non‐transgenic) through gene flow may favor the persistence and dominance of the weedy population in the production fields (Lu et al., 2016). Effective field management of the hybrids containing a herbicide‐resistance trait (obtained via gene flow) can be a challenge if management relies on that specific herbicide. Gene flow transferring a herbicide‐resistant trait from cultivated rice to weedy rice was demonstrated by Shivrain et al. (2007). Another popular example is the transfer of herbicide resistance from cultivated Brassicas to their weedy relatives (e.g., Allainguillaume et al., 2006; Legere, 2005; Warwick et al., 2008). In grain sorghum, a number of herbicide‐resistant traits (non‐transgenic) have already been commercialized and some more are in the pipeline. Thus, the transfer of herbicide resistance through *S. bicolor* × *S. halepense* hybridization is likely to occur under practical field conditions, and such trait movement is expected to complicate field management (Kershner, 2010; Ohadi et al., 2017).

The potential for hybridization between *S. bicolor* × *S. halepense* observed here has significant ecological consequences. Gene flow involving sorghum as the female parent has significance in regards to feral sorghum, that is, sorghum establishing on roadsides and other non‐cultivated habitats as a result of seed dispersal during commodity transport and other means (Ohadi et al., 2017, 2018). Sorghum is a highly drought‐tolerant species, which is able to grow even in marginal environments such as the roadsides (Ohadi et al., 2018). Considering that 25% of the feral sorghum established on the roadsides from seed spill are expected to be male sterile, and that outcrossing potential is significantly higher with male sterile sorghum due to a lack of pollen competition, significantly higher frequencies of outcrossing are expected between *S. bicolor* and *S. halepense* in the roadside environments. The fact that *S. halepense* is a troublesome weed (Klein & Smith, 2021) suggests that the hybrids between *S. bicolor* × *S. halepense* are expected to be more robust and vigorous in the natural environments, having the productive traits of *S. bicolor* combined with the weediness traits of *S. halepense* (anecdotal evidence), although the phenotypic characteristics of the F_1_ hybrids obtained in this study need to be investigated thoroughly for making specific inferences. In fact, crop sorghum alleles can be frequently found in roadside johnsongrass populations, perhaps effectively contributing to their persistence. Morrell et al. (2005) found crop sorghum‐specific alleles in up to 32.3% of the individuals in adjacent johnsongrass populations occurring in roadsides and other natural areas in Texas and Nebraska. In lettuce, Hoofman et al. (2007) have shown that lettuce‐wild hybrids were more vigorous, with improved offspring fitness than the wild parent (Hoofman et al., 2009). The authors are currently investigating the F_1_ progeny characteristics and fitness in *S. bicolor* × *S. halepense* hybrids.

It is likely that the hybrids produced with feral *S. bicolor* in the roadside environments could in turn establish feral hybrid populations, with the ability of backcrossing with either of the parents occurring in these habitats, eventually leading to trait introgression (Ohadi et al., 2017). The establishment, persistence, and introgression of traits in feral oilseed rape in roadside environments have been widely reported (e.g., Bailleul et al., 2016; Crawley & Brown, 2004; Knispel et al., 2008; Sohn et al., 2021). In a study involving *S. halepense* × *S. bicolor* (*S. halepense* as the female parent), Arriola and Ellstrand (1997) compared the F_1_ hybrids with the *S. halepense* parent and observed that the hybrids did not differ from *S. halepense* in terms of tiller production, time of flowering, time to panicle production, pollen viability, fecundity, or biomass. *S. halepense* × *S. bicolor* hybridization (*S. halepense* as the female parent) is beyond the scope of the current study, but the authors have recently initiated experiments to investigate the nature of *S. halepense* × *S. bicolor* hybridization and the characteristics of the F_1_ progeny resulting from such hybridization.

Arriola and Ellstrand (1997) suggested that novel traits that are either neutral or beneficial are likely to persist in the wild populations. Nevertheless, alleles that provide a selective advantage are more likely to establish and increase in frequency in a wild population, compared to the neutral alleles (Lee & Natesan, 2006). Novel traits (transgenic or non‐transgenic) that can be beneficial for feral populations and the crop‐wild hybrids include but are not limited to, drought tolerance, salinity tolerance, insect pest and disease resistance, and high biomass production (Bagavathiannan & Van Acker, 2008). Transgenic sorghum lines with high biomass production traits are currently under development (e.g., Do et al., 2006). High biomass in plants is generally associated with high fecundity (Younginger et al., 2017). Conversely, certain forage quality factors (i.e., reduced lignin concentrations) may actually reduce biological fitness as lignin content is generally associated with insect tolerance (Liu et al., 2018). Herbicide resistance, however, is not a fitness‐enhancing trait. From an ecological standpoint, the transfer of herbicide resistance per se is not expected to influence the adaptive evolution of hybrids containing the resistance trait in the absence of the herbicide (Mallory‐Smith & Zapiola, 2008). Nevertheless, as discussed above, the use of the specific herbicide for the management of the hybrids can offer an advantage over the surrounding vegetation.

The rates of gene flow reported here are significant. However, even the upper levels of outcrossing determined in this study (maximum 1.762%), across the different *S. bicolor* genotypes, pollen load, and environments, are substantially lower than previously thought, especially under conditions of pollen fertility in *S. bicolor* (maximum 0.49% under male fertility). Findings therefore suggest that *S. bicolor* × *S. halepense* hybridization can be managed through sound genetic mitigation and field stewardship measures. Given that the *S. bicolor* lines evaluated here are used directly as seed parents in commercial hybrids and as breeding parents to create new parental lines, this information indicates that outcrossing can be mitigated greatly by the selection of appropriate seed parents for hybrid sorghums (Pfeiffer et al., 2019).

Results also show that pollen load plays an important role in outcrossing rates and scenarios leading to the occurrence of male‐sterile *S. bicolor* plants around *S. halepense* should be addressed. Management should thus include the elimination of volunteer and feral *S. bicolor* in field and roadside situations. Moreover, this research was conducted in a field with extremely high densities of *S. halepense* infestation, simulating a worst‐case scenario. In fields with low to moderate densities of *S. halepense*, the pollen load from this species is expected to be substantially lower, resulting in much lower outcrossing frequencies across an entire field. Thus, robust integrated management programs should be implemented to keep *S. halepense* population densities at low levels, which in turn will help minimize the risk of outcrossing. A thorough gene flow mitigation and stewardship plan needs to be developed and strictly followed, and findings from this research will greatly contribute to such a document. For instance, a field management stewardship guideline for herbicide resistance mitigation in herbicide‐tolerant grain sorghum production has been recently developed by the United Sorghum Checkoff Program (Bean et al., 2022), which incorporates the findings reported here.

It is important to note that the current research only investigated gene flow with *S. bicolor* as the seed parent. The nature and dynamics of gene flow involving *S. bicolor* as the pollinator parent is not well understood and are currently being studied by the authors. Moreover, the weediness and persistence of the hybrid progenies also dictate the long‐term consequences of hybridization between these two species. Hoofman et al. (2008) showed through simulation modeling that wild relative parent can be displaced in natural populations by more vigorous hybrids, depending on the fitness characteristics of the hybrid progeny. The authors are currently investigating the adaptive traits of the F_1_ progeny resulting from *S. bicolor* × *S. halepense* hybridization in parallel experiments. The stewardship plans should consider all these aspects for long‐term effectiveness and sustainability.

## 5 CONCLUSIONS

The rate of *S. bicolor* × *S. halepense* hybridization was governed primarily by the frequency of 2n female gamete formation in *S. bicolor*, which differed among *S. bicolor* genotypes evaluated herein. The presence of *S. bicolor* pollen decreases interspecific hybridization frequencies by up to two orders of magnitude. These results indicate that gene flow can be mitigated greatly by selecting *S. bicolor* genotypes that are less likely to hybridize with *S. halepense*. The vast majority of the interspecific hybrid progeny were tetraploids; only a few triploids and an occasional pentaploid and hexaploid were recovered. Among these interspecific hybrids, the phenotypes manifested as taller than both parents with leaf widths and stem diameters that were intermediate to the parents. The confirmation of significant levels of gene flow between *S. bicolor* and *S. halepense* may have broader ecological implications, in addition to the agronomic consequences on successful weed management. Such consequences, especially in the long term depend largely on the hybrid fitness characteristics. Ongoing research by the authors will establish the fate of the hybrid progeny under field conditions, fitness consequences, weediness potential, and long‐term dynamics. This study was focused on interspecific hybridization when *S. bicolor* served as the female parent; it is important to assess hybridization rates when *S. bicolor* is the pollinator parent. Furthermore, research on gene flow mitigation through the management and genetic means and developing suitable stewardship protocols are needed. By further understanding, the biology of these crop‐weed systems, more conscious decisions can be made regarding the stewardship of novel sorghum technologies, including herbicide‐resistance traits.

## CONFLICT OF INTEREST STATEMENT

The authors declare that no conflict of interest exists.

## BENEFITS GENERATED

Benefits generated from this research accrue from the sharing of our data and results to the public.

## Data Availability

Data for this study are available at: to be completed after manuscript is accepted for publication.
